# Cigarette Relighting: A Series of Pilot Studies Investigating a Common Yet Understudied Smoking Behavior

**DOI:** 10.3390/ijerph18126494

**Published:** 2021-06-16

**Authors:** Carolyn J. Heckman, Olivia A. Wackowski, Rohit Mukherjee, Dorothy K. Hatsukami, Irina Stepanov, Cristine D. Delnevo, Michael B. Steinberg

**Affiliations:** 1 Cancer Prevention and Control Program, Cancer Institute of New Jersey, Rutgers University, New Brunswick, NJ 08901, USA; 2 Center for Tobacco Studies, Rutgers University, New Brunswick, NJ 08901, USA; wackowol@cts.rutgers.edu (O.A.W.); rm930@sph.rutgers.edu (R.M.); delnevo@cts.rutgers.edu (C.D.D.); steinbmb@rwjms.rutgers.edu (M.B.S.); 3 Department of Psychiatry, University of Minnesota, Minneapolis, MN 55454, USA; hatsu001@umn.edu; 4 Masonic Cancer Center, University of Minnesota, Minneapolis, MN 55454, USA; stepa011@umn.edu; 5 Department of Medicine, Rutgers Robert Wood Johnson Medical School, New Brunswick, NJ 08901, USA

**Keywords:** cigarettes, relighting, smoking

## Abstract

Background: The act of extinguishing, saving, and later relighting unfinished cigarettes is a common yet understudied behavior that may have implications for tobacco treatment and health. Methods: This paper presents four pilot studies investigating various aspects of this topic: (1) the prevalence of relighting among NJ and NY Quitline callers (*n* = 20,984); (2) the prevalence and correlates of relighting in two national surveys (*n* = 1008, *n* = 1018); (3) a within-subject (*n* = 16) laboratory experiment comparing cigarettes smoked per day and exhaled carbon monoxide when relighting and not relighting cigarettes; and (4) a national survey of tobacco treatment providers’ (*n* = 150) perceptions of relighting. Results: Relighting was found to be common (approximately 45% of smokers), and associated with lower socioeconomic status, heavier smoking and nicotine dependence, greater smoking-related concerns, as well as high levels of exhaled carbon monoxide. Providers noted the potential importance of relighting but reported that they do not regularly incorporate it into their assessment or tobacco treatment planning. Conclusions: These findings address a major research gap in the emerging research on this common behavior. Future research is needed to better understand the potential implications of relighting for policies and clinical practices related to tobacco cessation and health.

## 1. Introduction

Cigarette smoking remains the leading cause of preventable death in the United States. Although smoking prevalence has decreased in recent years, approximately 34 million Americans continue to smoke [1]. Smoking behaviors vary among individuals and could impact health outcomes and cessation. One type of understudied smoking behavior is the act of extinguishing, saving, and later relighting unfinished cigarettes (“relighting”). Prior studies with clinical and local samples suggest that the prevalence of ever or sometimes relighting unfinished cigarettes ranges from 44% to 73% of smokers [2,3,4,5,6,7]. Thus, while tens of millions of Americans may be relighting, very little research has focused on this topic.

The few studies that have investigated relighting found associations with characteristics related to tobacco disparities including being older, African American or Hispanic, unemployed, with lower income, lower education status, an unstable living environment, or being sick or disabled [2,3,5,7,8]. Relighting has also been related to smoking menthol cigarettes [8], which may allow smokers to inhale smoke and toxicants more deeply into their lungs, due to the anesthetic effects of menthol. It is known that menthol cigarettes are marketed to and preferred by Black smokers [9].

The findings from traditional smoking topography and compensation studies suggest differences in how relit versus first-lit cigarettes are smoked. When smoking two cigarette halves in two episodes (as can occur with relighting a saved cigarette), smokers may smoke more intensely and completely than when smoking one cigarette during one episode [10,11]. Such compensatory behavior may be associated with increased toxicant exposure, such as carbon monoxide (CO) [12]. Higher levels of toxicants (tar [13], nicotine [10,14], formaldehyde (an IARC carcinogen) [15], nicotine-derived *nitrosamine* ketone (NNK), and N-nitrosonornicotine (NNN) (carcinogenic tobacco-specific nitrosamines [TSNA] [16]) are produced from half-smoked or proximal halves of cigarettes compared to unburned or distal halves. This may lead to greater toxicant exposure from the same number of cigarettes per day (CPD) when relighting.

Increased toxicant exposure may contribute to an elevated risk for adverse health effects. A case-control study of 207 individuals from Nepal found a strong association between relighting and lung cancer in analyses that were unadjusted (OR = 4.47, CI = 2.28–8.78) and adjusted (OR = 37.63, CI = 7.55–187.46) for 17 demographic and health risk factors [17]. Two early studies with English men over 40 also found associations between relighting and lung cancer or chronic bronchitis. In the first study, 40.4% of 438 men who relit developed lung cancer compared with 27.8% of 562 non-relighters (*p* < 0.001) [4]. In the other study, of 1501 male relighters, those who usually relit had significantly higher prevalence of chronic bronchitis (39.7%) compared with those who did not usually relight (32.9%) [6]. Even these crude estimates illustrate the potential impact of relighting, with one author hypothesizing that greater chronic bronchitis may be due to “extinguishing and relighting of charred and possibly compressed tobacco” that leads to “a greater production of those substances in smoke” [6].

Given what little research exists on this emerging research topic, a better understanding of this phenomenon is critically needed to lay the groundwork to assess its potential health impact and inform interventions for providers and tobacco users in the future. This series of studies, conducted in the US, explores several aspects of relighting cigarettes: (1) the prevalence of relighting among tobacco treatment seekers; (2) the prevalence and correlates of relighting; (3) effects of relighting cigarettes on exhaled CO—a proxy for potential increased toxin exposure; and (4) relighting assessment and its perceived clinical importance among tobacco treatment providers. These studies will provide necessary foundational information on this important behavior for future research related to policy and practice on this topic.

## 2. Materials and Methods

### 2.1. Relighting among Utilizers of a Tobacco Treatment Quitline

In order to learn about the frequency of relighting among treatment-seeking smokers, data from the first study are based on a large sample of smokers calling into two state-based smoking cessation resources (Quitlines). During a 6-month period (August 2015–February 2016), a question about relighting cigarettes was added to the initial assessment tool used to screen smokers calling in to the New York and New Jersey state telephone Quitlines. Specifically, smokers were asked “How often do you relight cigarettes?” (always, often, sometimes, rarely, never). Callers to the Quitline were categorized as “relighters” (always, often, sometimes) or “not relighters” (rarely, never). The percentage of callers during that period who responded to how frequently they relight cigarettes was determined.

### 2.2. National Survey Data Collection—Prevalence and Correlates of Relighting

Additional data about the prevalence and frequency of relighting is presented from two national online surveys of adult (ages 18+) smokers. In both studies, smokers were recruited by the research company Ipsos (formerly GfK) from its national research panel (*KnowledgePanel*), assembled through a probability-based sampling of addresses from the US Postal Service’s Delivery Sequence File. In addition, smokers were defined in both studies as those who have smoked 100 cigarettes in their lifetime and now smoke “everyday” or “some days”. Additional eligibility criteria for the first survey (2016) included not currently using smokeless tobacco. Ipsos panel members can enter raffles or sweepstakes for cash rewards and other prizes.

The first survey was conducted in 2016 with 1008 cigarette smokers aged 18 years or older. GfK sampled 2654 participants, and 1288 (48.5%) completed eligibility questions. Of these, 1008 qualified for and completed the survey online. The relighting item asked participants “How frequently, if at all, do you “butt-out” your cigarette and save it to re-light and smoke later?” (never, rarely, sometimes, often, always). The participants were also asked how often they thought about smoking health risks, with the same response options. The proportion of the sample reporting relighting “sometimes” and frequently (i.e., “often” or “very often”) were calculated.

The second survey was conducted in 2021 (January–February) with 1018 adult smokers. IPSOS sampled 1852 participants, and 1171 (63%) completed eligibility questions. Of these, 1018 qualified for and completed the survey online. A relighting item asked participants “How often, if at all, do you “put-out” a cigarette and then relight it and smoke it at a later time?” Response options were made more specific than in the prior survey: at least once a day; several times a week; about weekly; once in a while; and never. Relighting “frequently” here was defined as at least weekly relighting. Smokers who responded “at least once a day” were also asked “About how many of the cigarettes you smoke in a day do you relight?” (less than half of them; about half of them; most or all of them). Descriptive statistics were conducted for both datasets and chi-square tests were conducted to examine associations between relighting frequency in 2021 and smoker demographics and other smoking history/behavior variables (with significance levels of *p* < 0.05). For these bivariate tests, we collapsed the relighting frequency categories to relighting: (1) at least once a day; (2) about weekly or several times a week; and (3) once in a while or never.

### 2.3. CO among Relighters—Proxy for Toxin Exposure and Potential Harm

A within-subject pilot study was conducted in 2019 with 16 adult current smokers of 10 or more cigarettes per day who reported relighting cigarettes. The participants attended a baseline session where demographics and questionnaire data were collected. Baseline measurements of exhaled CO levels before and after smoking a full cigarette were collected. For the next 24–48 h, participants were instructed to smoke only the first half of their own brand of cigarette, which had been marked at the half-way point, and then extinguish and save the second half. In the final 24–48 h period, the participants were instructed to smoke only the previously smoked halves that had been saved. The participants returned to the lab for exhaled CO measurements once a day at a similar time of day. At the visit, CO levels were measured immediately prior to and immediately after smoking one of the cigarette halves. The participants were asked to smoke their typical amount during the study periods and encouraged not to relight cigarettes during the first-lit period. However, any cigarettes that were relit during this period were tracked via a daily log. Paired *t*-tests were used to compare CPD and exhaled pre- and post-smoking CO levels at baseline versus first-lit or relit cigarettes.

### 2.4. Relighting Inquiries among Tobacco Treatment Providers

In order to learn what providers, who potentially see a large number of smokers, perceive about relighting and whether it affects their practice, a sample of tobacco treatment providers were surveyed. 150 Certified Tobacco Treatment Specialists (CTTS), who previously participated in an accredited tobacco treatment training program, were surveyed via an on-line survey instrument during July–August 2017. The survey collected information about the tobacco treatment specialist, as well as their beliefs and experiences regarding the tobacco dependence treatment they deliver. Specifically, participants were asked if they inquire about their patients’ cigarette relighting behaviors during assessment, and if they do, they were asked for reasons why patients relight their cigarettes and if assessing relighting during treatment would be clinically useful.

## 3. Results

### 3.1. Relighting among Utilizers of a Tobacco Treatment Quitline

Among 20,984 callers to the NJ/NY quit-lines over a 6-month period, 9371 (45%) reported relighting at least sometimes, and 4385 (21%) of callers reported frequent relighting (often or always).

### 3.2. National Survey Data Collection—Prevalence and Correlates of Relighting

The incidence of relighting *sometimes* or *once in a while* was 24–31% (see Table 1). Relighting *frequently* (i.e., often, very often, or at least weekly) was 21–39%. Relighting *daily* was 23% in 2021. Among daily relighters in 2021, about 56% reported relighting *at least half of their CPD*. In 2016, daily smokers who frequently *thought about smoking health risks* had a higher prevalence of frequent relighting (23%) than those who reported never or rarely thinking about smoking health risks (13%). In 2021, relighting was significantly more frequent among women, non-White and non-Hispanic smokers, those with less than a high school education, and those with lower household income levels (see Table 2). Relighting frequency was also significantly associated with daily smoking, being a menthol smoker, smoking within five minutes of waking (an indicator of nicotine dependence) and having used an e-cigarette in the past 30 days (Table 2). It should be noted that these surveys were not designed to study relighting and included only 1–2 items about it.

### 3.3. CO among Relighters—Proxy for Toxin Exposure and Potential Harm

When instructed to smoke half cigarettes (either first-lit or relit), the participants smoked fewer total cigarettes than in the 24 h before the baseline visit, when smoking mostly full cigarettes as usual. The participants smoked approximately twice the total number of cigarettes per day in the baseline state (M = 18.1, SD = 8.5) (mostly full cigarettes), compared to smoking first-lit (8.8 [3.5]) or relit (8.3 [3.8]) cigarettes (ps = 0.03). However, exhaled CO levels were not significantly different from baseline when smoking half-cigarettes (first-lit or relit) either prior to smoking in the lab (Pre-Smoking CO) or after smoking a half cigarette *in the lab* (Post-Smoking CO) (Table 3).

### 3.4. Relighting Inquiries among Tobacco Treatment Providers

Of the CTTS surveyed, 109 (73%) asked patients about relighting at least sometimes. Among those respondents, patients’ cited reasons for relighting were to: save money, cut back to quit smoking, reduce harm, and a lack of time to finish cigarettes in one sitting. Eighty-four percent of the providers agreed that knowing a patient’s relighting status would be useful in developing an appropriate tobacco treatment plan, but only 43% usually/always assessed relighting.

## 4. Discussion

Cigarette relighting is a potentially important public health problem considering its prevalence and potential impact on health and cessation. The proportion of smokers who report relighting in national surveys and two-state Quitlines is nearly half, with just under a quarter reporting doing so frequently or daily. This translates into tens of millions of smokers across the country engaging in this behavior. It is important to note that relighters are more likely to be of lower socioeconomic status and heavier, more nicotine dependent smokers. Some of the more common reasons reported for relighting cigarettes are to save money, cut back to quit smoking, reduce harm, or lack of time to finish cigarettes. Certified tobacco treatment specialists also believe relighting is important but tend not to incorporate it regularly into their assessments and treatment plans.

We also conducted a pilot study in which participants were instructed to smoke only half sections of their usual cigarettes (the first halves on one day and then the second halves on another day, by way of relighting them). We found that despite smoking fewer CPD during these study days, participants’ CO levels were similar for first-lit and relit halves compared to their CO levels at baseline after smoking full cigarettes. The limitations of our study were the small sample size and lack of session order counterbalancing. Additionally, study half cigarettes were stored in zip-lock bags instead of a cigarette pack in order to attempt to keep them “fresh”, but participants disliked them due to perceived “staleness”. Thus, we would anticipate that outside the lab, participants would smoke even more non-relit and relit half cigarettes.

In a prior study, we similarly demonstrated that although relighters smoked significantly fewer total CPD than non-relighters (16 vs. 20), they demonstrated similar exhaled CO values (20 vs. 19 ppm) [8]. This is consistent with findings from another study which found that the 7-day point-prevalence abstinence rates at 6-months for individuals in a tobacco treatment program were 30% in non-relighters and 19% among relighters, suggesting that cigarette relighting may lead to poorer cessation outcomes [Steinberg, unpublished data]. However, compared to our findings, one other study examining relighting showed different results. In a study of 172 relighters, Allen and colleagues found no significant associations between relighting and CPD, CO, cotinine, or biomarkers of oxidative stress [2]. It should be noted that their analyses did not account for recency of smoking/relighting, control for relevant variables such as nicotine dependence or socioeconomic status, and power may have been limited, particularly for heavy smokers or frequent relighters.

Although this series of studies investigated relighting from several different perspectives, these brief studies have some limitations including the relatively small sample sizes in some of the studies, the lack of control groups, the lack of assessment of the timing of the most recent cigarette for CO measures, and the use of convenience samples. The survey of Quitline callers may represent treatment seekers and not other smokers. The Ipsos panel is a large national panel cost-efficiently providing high accuracy and sample representativeness of the US population. Ipsos’s panel offers reduced susceptibility to sampling errors caused by non-probability methods of recruitment—a notable criticism of “opt-in” web-based or online panels- or coverage error due to excluding households without landlines or internet. However, data weighting is necessary when cell sizes for certain participant characteristics are small. Additionally, the language of the two national surveys of smokers was not identical, which may have affected the findings. Despite these limitations, the evidence presented in this series adds to the scant information on cigarette relighting and could be useful and hypothesis-generating for future studies in this area. For example, additional potential psychosocial correlates from health behavior theories and self-reported reasons for relighting among smokers would be informative. More rigorous toxicant and health studies using additional clinically relevant biomarkers and outcomes would be useful. Observation of treatment and cessation outcomes in relation to relighting would also be important.

## 5. Conclusions

Relighting was found to be common and associated with smoking-related concerns as well as high levels of exhaled carbon monoxide. Providers noted the potential importance of relighting but that they do not regularly incorporate it into their assessment or tobacco treatment planning. These findings address a major research gap in the emerging research on this common behavior. Screening for relighting, better understanding of the reasons for relighting, and educating providers about the potential impact of relighting on treatment and cessation may offer opportunities for intervention. Future research is needed to better understand the patterns of this behavior, how to measure it, and the potential policy and clinical implications of relighting for tobacco cessation and health.

## Figures and Tables

**Table 1:** Frequency of relighting behaviors among current adult smokers in two national US surveys.

**Frequency of Relighting, 2016 Survey (*n* = 1008)**	**%**
Very often	5.3
Often	15.4
Sometimes	24.0
Rarely	27.9
Never	27.5
**Frequency of Relighting, 2021 Survey (*n* = 1018)**	
At least once a day	23.5
Several times a week	12.9
About weekly	2.7
Once in a while	30.8
Never	30.2

**Table 2:** Frequency of cigarette relighting behaviors by demographic characteristics and other smoking-related variables, 2021 survey data (*n* = 1018).

Characteristic	Frequency of Relighting Behaviors			
	At Least Once a Day	About Weekly or Several Times a Week	Once in a While or Never	*p*-Value *
	%	%	%	
**Sex**				
Male	20.1	13.4	66.5	0.001
Female	27.3	18.0	54.7	
**Age in years**				
18–29	24.1	19.0	56.9	0.656
30–44	22.8	13.9	63.2	
45–59	25.3	14.1	60.5	
60+	21.5	16.9	61.6	
**Race/Ethnicity**				
White, Non-Hispanic	21.4	14.3	64.2	<0.001
Black, Non-Hispanic	30.2	27.8	42.1	
Other, Non-Hispanic	38.5	19.2	42.3	
Hispanic	22.4	5.6	72.0	
2+ Races, Non-Hispanic	23.1	30.8	46.2	
**Education**				
Less than high school	25.8	25.8	48.4	<0.001
High school	28.6	13.3	58.2	
Some college	21.7	14.0	64.3	
Bachelor’s degree or higher	8.3	11.6	80.2	
**Annual household income**				
<$25,000	33.9	23.7	42.4	<0.001
$25,000–$74,999	24.9	14.5	60.5	
$ ≥ 75,000	13.2	10.4	76.4	
**Smoking frequency**				
Smoke daily	26.4	15.7	57.9	<0.001
Smoke some days	12.2	15.0	72.8	
**Smoke how soon after waking**				
Within 5 min	33.7	16.0	50.2	<0.001
From 6 to < 30 min	25.9	18.0	56.1	
From 30 min to 1 h	19.5	17.8	62.7	
More than 1 h	12.4	9.9	77.7	
**Usually smoke menthol**				
Yes	28.0	18.1	53.9	<0.001
No	21.0	13.1	65.9	
**Ever tried to quit**				
Yes	22.9	16.8	60.2	0.193
No	24.8	12.2	62.9	
**Plans to quit smoking**				
No plans to quit	22.5	15.4	62.1	0.082
Planning to quit in next 30 days	18.1	10.3	71.6	
Planning to quit in next 6 months	27.6	19.4	53.1	
Planning to quit in the future, beyond 6 months	24.5	15.2	60.4	
**Used e-cigarette in last 30 days**				
Yes	31.6	21.1	47.4	<0.01
No	22.5	14.7	62.8	

* Note: *p*-values determined by Pearson’s chi-square tests.

**Table 3:** Mean (SD) exhaled carbon monoxide at baseline and during relit and first-lit conditions.

Measurement	Baseline (Full Cigarettes)	First-Lit Halves	Baseline vs. First-Lit *p*-Value	Relit Halves	Baseline vs. Relit *p*-Value
Pre laboratory smoking CO	22.8 (17.1)	24.8 (14.5)	0.38	24.3 (14.0)	0.52
Post laboratory smoking CO	25.5 (17.8)	26.9 (15.0)	0.54	25.8 (14.0)	0.88

Notes: SD = standard deviation, CO = exhaled carbon monoxide level; *p*-values determined by paired *t*-tests.

## Data Availability

The data presented in these studies are available upon request from the corresponding author. The data are not publicly available.
